# *Crambled: *A Shiny application to enable intuitive resolution of conflicting cellularity estimates

**DOI:** 10.12688/f1000research.7453.1

**Published:** 2015-12-07

**Authors:** Andy Lynch

**Affiliations:** 1 Cancer Research UK Cambridge Institute, University of Cambridge, Cambridge, UK

**Keywords:** Cancer, Whole-genome sequencing, Cellularity, Copy number, Sub-clonality, Cell lines, Shiny, R, Bioconductor

## Abstract

It is now commonplace to investigate tumour samples using whole-genome sequencing, and some commonly performed tasks are the estimation of cellularity (or sample purity), the genome-wide profiling of copy numbers, and the assessment of sub-clonal behaviours. Several tools are available to undertake these tasks, but often give conflicting results – not least because there is often genuine uncertainty due to a lack of model identifiability.

Presented here is a tool, "Crambled", that allows for an intuitive visual comparison of the conflicting solutions. Crambled is implemented as a Shiny application within R, and is accompanied by example images from two use cases (one tumour sample with matched normal sequencing, and one standalone cell line example) as well as functions to generate the necessary images from any sequencing data set.

Through the use of Crambled, a user may gain insight into why each tool has offered its given solution and combined with a knowledge of the disease being studied can choose between the competing solutions in an informed manner.

## Introduction

The generation of whole-genome sequencing data to investigate tumour samples has become commonplace, thanks in particular to initiatives such as the International Cancer Genome Consortium (ICGC) ^1^. Among the analyses being applied to the data generated are investigations of copy number changes ^2^, structural variants ^3^, and sub-clonality ^4^. These analyses typically require the establishment of segmented copy number profiles for the sample, which in turn rely on estimating the degree of contamination of normal tissue in a sample and the mean ploidy (equivalently the depth of coverage associated with a particular copy number state or the increase in depth associated with an increment in copy number).

Many tools have been developed that allow for the estimation of one or more of sample purity, copy number profile and clonality. These include e.g. ABSOLUTE ( www.broadinstitute.org/cancer/cga/absolute) ^5^, ASCAT ( heim.ifi.uio.no/bioinf/Projects/ASCAT) ^6^, CloneHD ( github.com/andrej-fischer/cloneHD) ^7^, OncoSNP-SEQ ( sites.google.com/site/oncosnpseq/) ^8^ and QPure ( sourceforge.net/projects/qpure/) ^9^ (for a more complete review see Yadav and De. ^10^). There are real problems of model identifiability in performing this task, particularly when sub-clonal solutions are allowed, as any errors or discrepancies in the purity, segmentation or identification of mean ploidy can often be explained as sub-clonal behaviour (many of the issues are discussed in Lonnstedt *et al.* ^11^). Indeed, many tools acknowledge the inherent uncertainty in the calculations. As a consequence, applying two tools to a data set may return two conflicting solutions.

Contrasting this problem with another common analysis applied to whole-genome cancer sequencing data, that of identifying somatic single nucleotide variants and indels then there is one striking difference: SNV calls can be easily validated. This can be via a targeted experimental approach, or increasingly commonly through visual assessment using a tool such as the Integrative Genomics Viewer ^12^.

Herein is presented the *Crambled* tool to enable the visual assessment of the alternative purity/depth-of-sequencing solutions that can arise for a tumour sample. Through exploration of the solutions using *Crambled* one may achieve an appreciation of the reasons for each to have been offered. Coupled with knowledge of the tumour type, and the case history and pathology of the particular patient under examination, it may be possible to then select a preferred solution.

## Methods

### Implementation

***Overview*.** The *Crambled* tool is implemented as a Shiny ^13^ application in R ^14^. In essence it takes in a figure depicting the sequencing data (akin to a patchwork plot ^15^ or grid plot ^11^), constructs a second figure based on a solution for the cellularity/depth conditions, and displays them in a superimposed manner. By allowing for a dynamic adjustment of the cellularity/depth solution being considered, *Crambled* enables an intuitive comparison of competing solutions and a tactile exploration of the solution space that provides insight into why different tools offer different solutions and informs the user in choosing between them.

**Figure 1.**
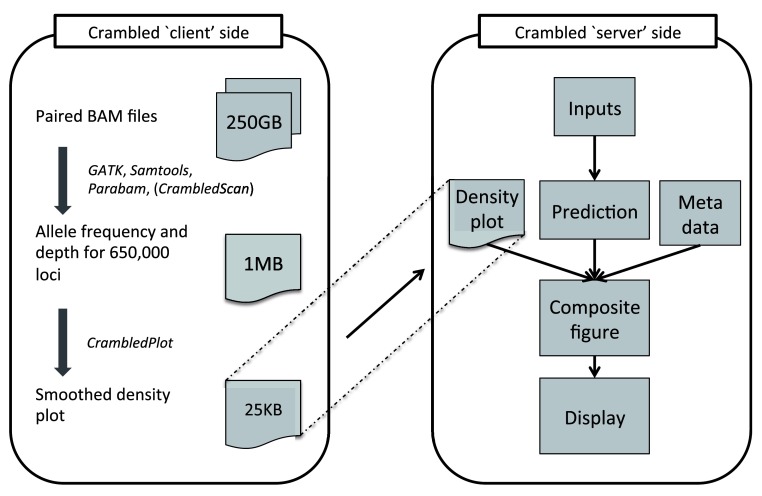
A schematic of the operation of the *Crambled* tool. Tasks are divided between those on the ‘client’ side (that aim to reduce the size of the data that need to be transferred) and the main application on the ‘server’ side (where the cellularity/depth solutions can be investigated).

The *Crambled* tool is divided into two sections as depicted in Figure 1, nominally a ‘client’ side and a ‘server’ side, to avoid having to upload across a network the large BAM files that contain the genome sequencing data.

Even when both ‘client’ and ‘server’ sides are run on the same machine, as is envisaged for a typical user, there are clear benefits to the division of labours. The initial data processing is an expensive operation and should only be performed once, with the output stored for potential repeated and spontaneous investigations in the future.

***‘Server’-side tools*.** The *Crambled* application consists primarily of a dialogue box for selecting an image representing a sequencing experiment, two sliders for specifying cellularity and the depth of a single copy (that is the depth associated with a single copy present in all cells in a sample or, equivalently, half of the depth of coverage in diploid regions), and a display that dynamically updates the selected image with superimposed predictions based on the chosen values.

The display figure is generated by first writing out the predictions to a temporary image before the two image files are combined. For two values, cellularity ( *C*) and single-copy depth ( *D*), the initial predictions for a region with *n*_1_ copies of one allele and *n*_2_ copies of the second are:

$$\text{Depth}=D\times \left[2\times \left(1-C\right)+\left(n_{1}+n_{2}\right)\times C\right]\;(1)$$

and

$$\text{Minor allele fraction}=\frac{1+C\times \left(n_{2}-1\right)}{2+C\times \left(n_{1}+n_{2}-2\right)}.\;(2)$$

**Figure 2.**
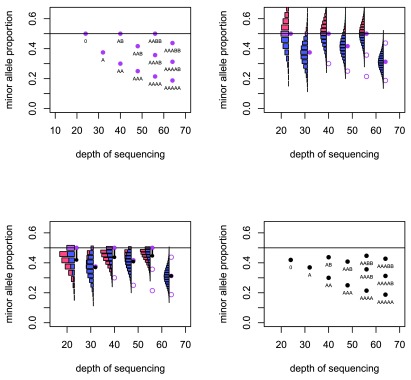
Deriving the predicted values used by *Crambled*. Top left: The basic predicted depths of sequencing and allele fractions that are expected for a number of copy number states. Top right: For six cases the uncertainty in the allele fraction that will be observed is illustrated. Bottom left: When the distribution folds at the 0.5 level, the expected value of the allele fraction is reduced. Bottom right: The predicted depths of sequencing and allele fractions that are used in the *Crambled* tool.

These values are depicted in Figure 2.

For a locus that is heterozygous in the germline sample, the observed minor allele fraction is recorded. If the SNPs are phased, and one is considering a region in allelic balance, then it is possible to take all the allele fractions from one allele and gain a mean fraction of 0.5. Without the phasing information, the minor allele will be taken at each locus and so the mean allele fraction recorded in the region will be below 0.5. Even in regions of strong allelic imbalance, it is possible that the observed allele fraction can exceed 0.5 when the true value does not, and so the mean allele fraction in those regions will also be biased.

Given the true minor allele fraction and depth, the observed fraction of the true minor allele has an estimable probability distribution. The mass of probability for allele fractions greater than 0.5 then ‘folds’ below 0.5 to reflect the distribution of the observed fraction of the observed minor allele, which is the value recorded. In this manner, the mean of the distribution is reduced. This effect is greater for small depths of sequencing due to the greater variance in observed allele fraction that comes from having a smaller denominator. The effect is greater also for allele fractions that are close to 0.5 ( Figure 2).

The final options available in the server interface allow the user to edit some of the metadata concerning the image loaded into *Crambled*. Specifically, the user can depict a different range of depths as appropriate for their experimental data, and can specify the size of the image if this is different to the default.

***‘Client’-side tools*.** It may be that the user will already have details of germline variants, and their depths and allelic fractions in the tumour sample, from which to create an image to load into *Crambled*. Such data may be purposefully sought from tools such as GATK ( www.broadinstitute.org/gatk/) ^16^, SAMtools ( www.htslib.org/) ^17^, or parabam ( parabam.readthedocs.org/). Alternatively, the information may be available as the side effect of running a somatic mutation caller. In the event that such data are not available, functions are supplied with *Crambled* to enable suitable data to be generated from BAM files.

The function CrambledScan() is accompanied by a file that lists (for Human Genome Issue 19) 177,299 sites that are highly likely to be heterozygous in a sample. This was generated from the “snp138Common” table for hg19 from the UCSC Table Browser ^18^. Rsamtools ^19^ is used to interrogate the BAM files at those locations. At a depth of coverage of about 40×, this typically returns approximately 80,000 heterozygous sites once quality filters have been put in place.

**Figure 3.**
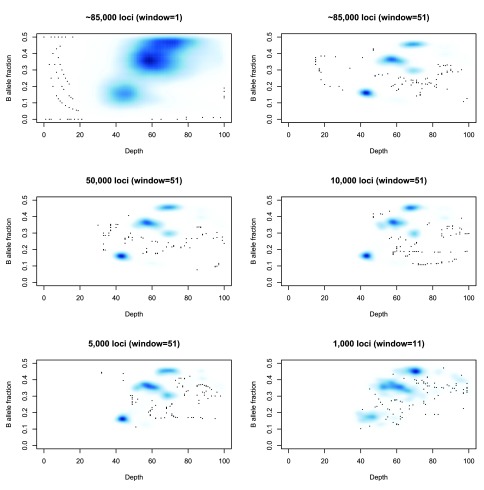
The capabilities of a small number of loci to capture the grid-plot structure. Top left: A smoothed density plot of depth and B-allele fraction for sample SS6003302 (see Use Case 1) using 84,252 loci and no running average (equivalently a running average with a window length of 1). Top right: The same density plot with a running average (window size: 51 loci) first applied to the depth and minor allele fractions. Middle left, Middle right, Bottom left: The same density plots produced from samples of 50,000, 10,000 and 5,000 loci respectively. Bottom right: The same density plot produced from a sample of 1,000 loci and using a window of 11 loci for the running average.

A running median is then applied to the depths and allele fractions at these sites to reduce noise. Note that this is not an attempt to characterize the entire genome, but merely to capture the cellularity information. Thus it does not matter if fine-grain copy number changes are lost in this smoothing. Ideally one would have as many germline heterozygous loci as possible (typically 2,000,000 such loci for an individual), but extracting them comes at a computational cost. The approximately 80, 000 loci used here will usually suffice for this limited task, and fewer may be feasible as seen in Figure 3.

The need for using a running average is also demonstrated in Figure 3. Note that as the number of heterozygous loci being used decreases, then a window of, *e.g.,* 50 loci represents a much larger genomic region, making it more likely that the values being averaged represent several distinct states and that the average will not represent a true state. Reducing that window will increase the noise in the picture and so a balance must be sought that reflects the complexity of the genome being studied.

The plotting function ( *CrambledPlot*) takes the output of this approach and produces a standard R plot, but with parameters set to values that the *Crambled* application will anticipate. Should it be necessary to change these, e.g. to increase the limits on the plotting area because a sample has been sequenced to 500× depth of coverage, then the *Crambled* app needs to be informed via the metadata input options.

### Operation

The tool and code presented here are built on top of R ^14^ and Shiny ^13^, and thus will run on a large number of operating systems. The dependencies within R are on the ‘Shiny’ package (obtainable from The Comprehensive R Archive Network [ cran.r-project.org]) to run the application, and the ‘Rsamtools’ packages from Bioconductor ^20^ required for the code to prepare images for *Crambled*. Naturally, if the BAM files have been aligned against a genome other than human genome issue HG-19, then the list of suggested loci for investigation that accompanies the *Crambled* tool will not be relevant and an alternative list must be generated. The user must create an image from the sequencing data of interest (either using the code provided, or independently in the style of the example images) and then load this into the *Crambled* application. The *Crambled* application can be run on a local machine that has R and the R shiny package installed, or it can be run on a Linux machine running the Shiny Server software ( www.rstudio.com/products/shiny/shiny-server/). It has been extensively tested on a Ubuntu 12.04.5 machine running R version 3.2.1, Shiny 0.12.2 and Rsamtools 1.20.5. and an Apple computer running OS X version 10.9.5, R version 3.1.2, Shiny 0.11.1 and Rsamtools 1.18.3.

The code provided with *Crambled* for creating the images takes approximately 20 minutes to run (using a single processor on a reasonable desktop machine) on a single 100GB cell line BAM file, or approximately an hour to run on two 150GB BAM files representing a tumour/normal pair.

## Use cases

### A tumour/normal pair

The first illustration of *Crambled* is an application to a previously published tumour/normal pair from an oesophageal adenocarcinoma (OAC) patient ^21^. Two estimates of cellularity have been suggested in this case: 81% and 68%. While a small variation in estimates is natural, in this case the difference is too great to put down to the (in)stability of the estimate and it warrants investigation with the *Crambled* tool.

The first step is to generate the image to load into the *Crambled* Shiny application. Assuming that one has obtained the two BAM files (here called “SS6003301.bam” and “SS6003302.bam”), within the R environment one types:



                        > 
                            **source**("crambledfunctions.R")> CrambledScan(normal="SS6003301.bam",tumour="SS6003302.bam",
                            **title**="SS6003302")
                    


**Figure 4.**
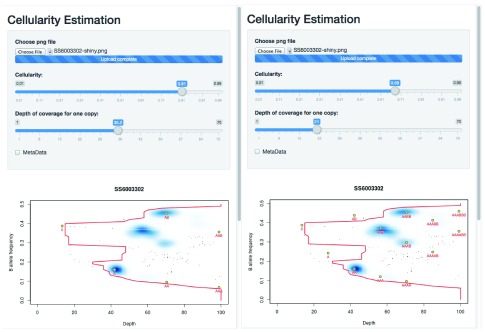
Resolving two competing solutions for a tumour/normal-tissue pair. Left: The solution at 81% cellularity fits the extremes of the data (the red contour) well, suggests a (hypo)diploid solution and leaves two states (blue regions in the density plot) unexplained by the clonal model (presumably representing sub-clonal behaviour). Right: The solution at 68% cellularity fits the extremes of the data equally well, and explains all of the main observed states, but suggests that the tumour is largely tetraploid.

This produces the file ‘SS6003022-shiny.png’ (available in the ExamplePlots folder at https://github.com/dralynch/crambled.git), that can be loaded into the *Crambled* application. The application may be operating on a server or, after installing Shiny, within R one can type:



                        > 
                            **library**(shiny)
> runApp("crambled_app/")
                    


to begin the tool. Uploading the figure, one can then test the two solutions as seen in Figure 4.

One can see that the solution with 81% tumour assumes that the sample is primarily diploid, with some regions of copy number loss and, crucially, some regions of data that lie off of the predicted grid and so must represent ‘sub-clonal’ states. By contrast, the 68% cellularity solution shows that all of the data lie on the predicted grid (i.e. there is little-to-no evidence of sub-clonality). However this solution suggests that the sample is broadly tetraploid.

Since OAC cases are often tetraploid, and this solution explains away all of the sub-clonality, the 68% cellularity solution seems to be the favourable one. While sub-clonal behaviour is also seen frequently in OAC, and the 81% solution is entirely valid, that the suggested sub-clonality conveniently only occurs at frequencies that can be explained by the tetraploid solution leads to a favouring of 68%.

### A cell line

Cell lines present a different problem. Typically one would not be trying to estimate the cellularity of a cell line (which should be pure), but resolving the depth-of-sequencing/copy number can still be an issue, as can confirming that only a single population of cells is present.

Commonly, there will be no germline reference sample for a tumour cell line. Coupled with this, due to the (near-)perfect purity of a cell line, it is not possible to distinguish a germline homozygous site from a heterozygous site that has undergone loss-of-heterozygosity (LOH). Nor is it possible to distinguish a clonal somatic mutation from a germline-heterozygous site.

For the purposes of inferring cellularity, neither of these confusions matters except that in producing a patchwork- or grid- plot the signal from sites with no heterozygosity will drown out any observations from the (more informative) loci that have gained or retained heterozygosity. The purity of the cell line, and the consequent separating of LOH-representing regions from other regions on the plot allows for separation of the two groups (with a simple threshold on allele fraction) and subsequent down-sampling of the LOH-like regions. Since this case requires different preparation, a separate command is provided for the creation of the plot.

This usage is illustrated using whole genome sequencing from the Genome Modelling System ( github.com/genome/gms) ^22^. Three lanes of whole genome sequencing data for the HCC1395 breast cancer cell line (gerald_D1VCPACXX_1.bam, gerald_D1VCPACXX_2.bam, and gerald_D1VCPACXX_3.bam) were downloaded (via github.com/genome/gms/wiki/HCC1395-WGS-Exome-RNA-Seq-Data) and aligned to the EnsEMBL release 71 assembly of the GRCh37 (hg19) human genome ( apr2013.archive.ensembl.org/index.html).



                        > 
                            **source**("crambledfunctions.R")> CrambledScanCellline(normal="gerald_D1VCPACXX.bam",
                            **title**="HCC1395")
                    


This produces the file ‘HCC1395-shiny.png’ (available in the ExamplePlots folder at https://github.com/dralynch/crambled.git). This image can be loaded into the *Crambled* application as per the previous use case. Therefore, within R one would type:



                        > 
                            **library**(shiny)
> runApp("crambled_app/")
                    


**Figure 5.**
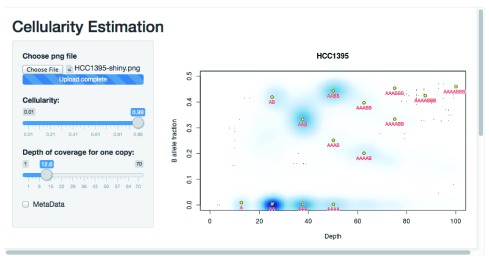
Illustrating the application to cell line data.

The results can be seen in Figure 5. The cell line appears to be a single population of cells, with copy numbers mainly in the 2 to 4 range (with some regions at a copy number of 1, and others at a copy number of 5 but with a four-to-one allele balance). Note that the thresholding of allele fractions may, at low depths, cause an artefactual data cloud to appear in the plot close to the threshold and suggestive of sub-clonality, but that this should be ignored.

## Discussion

The code provided to produce the images to load into the *Crambled* application suffices for the intended purpose, but has shortcomings with regard to other tasks. Firstly, it is prohibitively slow if one wishes to search for the complete set of informative loci in the genome, and the resolution that such a set brings to the problem is useful. One of the other tools mentioned in the introduction can be used if this is desired.

Secondly, there is no correction either for biases such as GC-related biases in the tumour sample, nor for regions of the normal sample that are polyploid, nor for artefactual mapping. Any of these would reduce the noise in the figures (particularly that about the depth estimate) and could potentially allow fewer loci, or a smaller window for the running average, to be used.

Finally, it should be noted that little effort is required to annotate the uploaded image more than is currently the case. Individual chromosomes could be picked out and highlighted using different colours. Alternatively, if one wanted to know whether e.g. TP53 had undergone LOH, it would be straightforward to mark the location of nearby SNPs and see where they lie in the plot.

## Summary

*Crambled* is a tool that allows for interactive investigation of the multiple solutions that are possible when inferring the cellularity (and related attributes) of a tumour sample that has undergone whole genome sequencing. Implemented as a Shiny application it can be used on most platforms and comes with example files and code to prepare such files from one’s own sequencing data.

## Software availability

1. Software available from: https://dralynch.shinyapps.io/crambled_app (limited usage server - the preference is for the user to download the *Crambled* application and run it locally)
2. Latest source code: https://github.com/dralynch/crambled.git
3. Link to archived source code as at time of publication: http://www.dx.doi.org/10.5281/zenodo.34147 ^23^
4. License: Lesser GNU Public License 2.0: https://www.gnu.org/licenses/old-licenses/lgpl-2.0.html
