# Supplementary material for: Crambled: A Shiny application to enable intuitive resolution of conflicting cellularity estimates
Source: F1000Res. 2015 Dec 7;4:1407. [Version 1] doi: 10.12688/f1000research.7453.1 (PMC4765721; doi:10.12688/f1000research.7453.1)
Supplement: Peer review history

# Peer review: Referee response for version 1

**DOI:** 10.5256/f1000research.8030.r12383

**Published:** 2016-02-19

**Authors:** Ingrid Lönnstedt

**Affiliations:** 1 Department of Immunology, Genetics and Pathology, Uppsala University, Uppsala, Sweden

The article describes "Crambled", an online application to investigate cellularity/ploidy solutions of genome sequencing copy number data. The easily accessible and soundly developed tool, enabling informed choices between potential solutions, is the main contribution of this study. It also comprises additional advances to existing methods, as (i) the naturally occurring bias in minor allele fractions of heterozygous loci is assessed and adjusted for, and (ii)  crambled suggests potentially heterozygous sites which can add substantially to the information a solution choice is based upon.

These are minor suggestions to improve the article:

- I would have enjoyed some performance statistics on Crambled, for example showing the fraction of samples tried that were successfully resolved, or the fractions of samples tried that had in practice a good fit to expected cluster centers for at least say 70% of the genome. At the moment I can only see the overall performance of a couple of of samples, and Figure 2 seems to be based on simulated data so I cannot assess whether this strategy works out well with real data.
- I have only tried the online Crambled application, not the full from bam file setup which requires source download. Based on the current version of the article, it is not obvious to me whether the full downloadable version of Crambled returns a list with the copy numbers of a chosen cellularity solution are assigned to genomic locations, or whether this solutions (which will commonly be a central desired outcome of the analysis) have to be extracted with a different software.
- In the last section before Discussion, I do not understand where the "cloud close to the threshold" is, which is falsely suggesting subclonality. Could that be explained better?
- The png figures to be supplied to Crambled should show "minor allele proportion" versus "depth of sequencing". Preferably explain that "depth of sequencing" is different from the definition of "Depth" in equation 1 (assuming you mean the total depth, not just the depth of one copy).
- I think many readers would have enjoyed some more instructions on how to play with the cellularity and depth in the application. What should I try to achieve, what should I try to avoid?

I have read this submission. I believe that I have an appropriate level of expertise to confirm that it is of an acceptable scientific standard.

# Peer review: Referee response for version 1

**DOI:** 10.5256/f1000research.8030.r11450

**Published:** 2016-01-05

**Authors:** Jan Oosting

**Affiliations:** 1 Department of Pathology, Leiden University Medical Center, Leiden, Netherlands

The manuscript by Lynch presents an R app ‘Crambled’ to ease the determination, in whole genome DNA sequencing experiments, of cellularity in tumour samples or to show the presence of sub clones in cellline samples.

A shiny app displays an image of the behavior of heterozygous SNPs according to read-depth and minor allele frequency, and overlays that with the theoretical positions of chromosomal aberrations at  user- defined cellularity and single copy coverage levels.

The image can be produced, starting from BAM-files, by the accompanying functions of the app.

- The manuscript is written clearly, and describes the functionality of the package well.
- I have not tested all functions of the package, but the R code is understandable and straightforward. The functions are commented throughout.
- As described in the paper, during the use of the shiny app, it is important to have an idea of the cellularity of the sample. Just playing around with the sliders does not converge to logical solutions easily. The example case 1 illustrates the usefulness of the app because it shows clearly that one of the cellularity solutions is to be preferred over the other.
- I would like more guidance on how to get the cellularity estimates. The references 5-9 seem to be geared mostly towards array type of data. It would be a good addition to provide a preferred solution in the workflow description.
- It would be good to mention the values for cellularity and single copy depth that were chosen for figure 2.

In conclusion, the manuscript presents a tool that is useful in the assessment of copy-number aberrations. There are a few minor details that could be improved.

I have read this submission. I believe that I have an appropriate level of expertise to confirm that it is of an acceptable scientific standard.
